# Dairy Cows’ Health during Alpine Summer Grazing as Assessed by Milk Traits, Including Differential Somatic Cell Count: A Case Study from Italy

**DOI:** 10.3390/ani11040981

**Published:** 2021-04-01

**Authors:** Giovanni Niero, Tania Bobbo, Simone Callegaro, Giulio Visentin, Cristina Pornaro, Mauro Penasa, Giulio Cozzi, Massimo De Marchi, Martino Cassandro

**Affiliations:** 1 Department of Agronomy, Food, Natural Resources, Animals and Environment, University of Padova, Viale dell’Università 16, 35020 Legnaro, Italy; g.niero@unipd.it (G.N.); tania.bobbo@unipd.it (T.B.); simone.callegaro@unipd.it (S.C.); cristina.pornaro@unipd.it (C.P.); mauro.penasa@unipd.it (M.P.); massimo.demarchi@unipd.it (M.D.M.); martino.cassandro@unipd.it (M.C.); 2 Department of Veterinary Medical Sciences, Alma Mater Studiorum University of Bologna, Via Tolara di Sopra 50, 40064 Ozzano dell’Emilia, Italy; 3 Department of Animal Medicine, Production and Health, University of Padova, Viale dell’Università 16, 35020 Legnaro, Italy; giulio.cozzi@unipd.it

**Keywords:** dairy cow, pasture, milk health-related trait, extensive farming

## Abstract

**Simple Summary:**

Dairy herds in alpine areas usually adopt summer grazing, mainly to reduce feeding costs. This practice is related to the maintenance of local traditions and to the manufacturing of niche dairy products. However, it is important to assess the impact of this practice on cattle health. This case study investigated how milk-related health traits vary across extensive grazing during the summer period, using data collected in a dairy herd whose cows were repeatedly controlled for individual milk samples. Although the transition from barn farming to pasture led to a reduction in milk production, proper grazing management can make dairy cows more resilient in terms of udder health and metabolic efficiency. Findings of the present research report suggested that pasture can be adopted to maintain dairy herd sustainability without impairing animal health.

**Abstract:**

Extensive summer grazing is a dairy herd management practice frequently adopted in mountainous areas. Nowadays, this activity is threatened by its high labour demand, but it is fundamental for environmental, touristic and economic implications, as well as for the preservation of social and cultural traditions. Scarce information on the effects of such low-input farming systems on cattle health is available. Therefore, the present case study aimed at investigating how grazing may affect the health status of dairy cows by using milk traits routinely available from the national milk recording scheme. The research involved a dairy herd of 52 Simmental and 19 Holstein × Simmental crossbred cows. The herd had access to the pasture according to a rotational grazing scheme from late spring up to the end of summer. A total of 616 test day records collected immediately before and during the grazing season were used. Individual milk yield was registered during the milking procedure. Milk samples were analysed for composition (fat, protein, casein and lactose contents) and health-related milk indicators (electrical conductivity, urea and β-hydroxybutyrate) using mid-infrared spectroscopy. Somatic cell count (SCC) and differential SCC were also determined. Data were analysed with a linear mixed model, which included the fixed effects of the period of sampling, cow breed, stage of lactation and parity, and the random effects of cow nested within breed and the residual. The transition from barn farming to pasture had a negative effect on milk yield, together with a small deterioration of fat and protein percentages. Health-related milk indicators showed a minor deterioration of the fat to protein ratio, differential SCC and electrical conductivity, particularly towards the end of the grazing season, whereas the somatic cell score and β-hydroxybutyrate were relatively constant. Overall, the study showed that, when properly managed, pasture grazing does not have detrimental effects on dairy cows in terms of udder health and efficiency. Therefore, the proper management of cows on pasture can be a valuable solution to preserve the economic, social and environmental sustainability of small dairy farms in the alpine regions, without impairing cows’ health.

## 1. Introduction

Low-input dairy livestock systems usually adopt pasture grazing in specific periods of the year, particularly in spring and summer; this is primarily dictated by pasture availability in those periods and thus by economic reasons [1,2,3,4]. Mountainous dairy farmers traditionally keep both replacement heifers and milking cows indoors during the cold months of the year to subsequently allow the herd to access the pasture when climatic conditions are more favourable. This practice is important for dairy farmers, and it has economic, social, historical, environmental and touristic implications [5,6,7]. Moreover, cheese manufactured with milk from grazing cows has peculiar flavour compounds, is richer in functional nutrients and is perceived by consumers as healthier and more sustainable; these aspects increase the value of the final product [8,9].

Grazing has positive implications on animal welfare, especially in terms of reduced cortisol and increased serotonin levels [4]; animal behaviour, such as lying and resting time [10,11]; and reduced aggressions [12]. Pasture is also beneficial for animal health because grazing cows tend to have lower incidence of lameness [13], hock lesions [14,15] and mastitis [16] compared to confined animals. Nevertheless, grazing may be a risk factor of gastrointestinal parasites infections [17]. Moreover, dairy cows may experience a negative energy status when exposed to grazing, which is reflected in a reduction in milk yield [5] and loss of body condition; therefore, a common practice is to provide concentrate supplementation to maintain the milk production of the herd and body fat reserves [18].

The use of rapid and cost-effective techniques, such as mid-infrared spectroscopy, [19] to monitor milk quality is very common for large-scale phenotyping within the official national milk recording scheme. Mid-infrared prediction models have been developed for major milk compounds (e.g., fat, protein and lactose contents) in the past and more recently for a series of detailed milk components and characteristics, including protein fractions [20,21]; fatty acids [22,23]; milk coagulation properties [24,25]; and animal health-related traits such as β-hydroxybutyrate (BHB), acetone and urea content [26,27]. Those phenotypes, coupled with the information provided by indicators of udder health, such as total somatic cell count (SCC), can be exploited by dairy farmers as predictors of changes in cows’ homeostasis and thus to take preventive actions to guarantee a high standard of animal health and maintain herd productivity. In addition, the recent implementation of new milk-testing technologies has allowed the routine recording of differential somatic cell count (DSCC), which is the ratio of neutrophils plus lymphocytes to the total SCC [28]. The novel DSCC trait, in combination with traditional SCC, provides a more precise indication of dairy cows’ udder health status and helps farmers identify healthy cows from those susceptible to the disease or with acute or chronic mastitis [29].

However, little is known about the effect of grazing in small dairy farms operating in mountainous marginal areas on milk health-related traits. Therefore, the objective of this trial was to investigate how and to what extent the health of dairy cows reared in an alpine herd was affected by summer grazing, by using milk traits included in the national milk recording scheme. Findings from the current case report should stimulate the exploitation of routinely collected milk traits to monitor cows’ health and welfare in other types of dairy herds operating in the Alps to increase the robustness of the presented results.

## 2. Materials and Methods

### 2.1. Herd

All the experimental procedures used in the trial were not invasive and, therefore, did not require the authorisation of the animal welfare committee. The commercial herd involved in this case study was located in Veneto Region (Pian del Cansiglio, Belluno, Italy) at 1100 m above sea level and was subjected to the official milk recording system. The experimental trial comprised 80 lactating cows: 55 Simmentals (SI), 3 Holsteins (HO) and 22 HO × SI crossbreds (CR). During autumn, winter and early spring, cows were kept indoors in a free stall barn and fed a total mixed ration. Milking took place twice per day, in the morning (5:00 a.m.) and in the afternoon (5:00 p.m.). Before the onset of the pasture season, cows received a total mixed ration, based on grass silages, hay, high moisture corn and a protein mix (Table 1). During late spring and summer, cows had access to the pasture adjacent to the barn. Cows grazed fresh herbage following the Voisin rotational grazing system [30], implying that animals utilised the entire herbage production of a paddock and moved to a different paddock every day. Milking took place in the same barn and at the same times of the day, as previously described. At the two daily milkings, cows received 2 kg of concentrate feed supplement made of high moisture corn and cereal meals.

### 2.2. Milk Sampling and Chemical Analysis

The experiment was designed to characterise animal production before and after the beginning of the grazing season (Day_0_). Repeated individual milk samples were collected from all the animals of the herd during the afternoon milking, between late May 2020 and late August 2020. The use of afternoon milking was chosen to avoid the bias and different accuracy in the quantification of milk yield due to alternate testing scheme, as reported by Cassandro et al. [31]. In particular, milk sampling was performed 1 and 3 days prior to the beginning of grazing (Day_−1_ and Day_−3_, respectively) to characterise the production of the cows in barn farming (BF) conditions. Similarly, milk samples were collected at 2, 3, 7, 10 and 14 days after the beginning of the grazing season (Day_+2_, Day_+3_, Day_+7_, Day_+10_ and Day_+14_, respectively) to characterise the production of the cows at an early grazing stage (EG). Moreover, milk samples were collected at 21, 49 and 91 days after the beginning of grazing (Day_+21_, Day_+49_ and Day_+91_, respectively) to characterise the production of the cows at the mid–late grazing stage (MLG).

Individual milk yield (MY, kg/milking) was recorded through the proportional sampler DeLaval MM6 (DeLaval Inc., Tumba, Sweden). Immediately after sampling, 200 μL of preservative (Bronopol; 2-bromo-2-nitropropan-1,3-diol) was added to 40 mL of milk and transferred at 4 °C to the laboratory of the Breeders Association of Veneto Region (Padova, Italy). Milk samples were warmed at room temperature, gently mixed by inversion 10 times to promote fat and solids homogenisation and analysed within 12 h for gross chemical composition (fat, protein, casein and lactose content; %), electrical conductivity (EC, mS), milk urea (MU, mg/dL) and BHB (mmol/L) using a MilkoScan FT6000 (FOSS Analytical A/S, Hillerød, Denmark). Fat to protein ratio (FPR) was calculated as the ratio of fat to protein content. Somatic cell count (n/µL) and DSCC (%) were determined using the new Fossomatic 7 DC (FOSS Analytical A/S). Skewness (17.26), kurtosis (356.96), and visual inspection of SCC distribution highlighted that the variable was not normally distributed. Therefore, values of SCC were log-transformed to the somatic cell score (SCS) using the formula SCS = 3 + log_2_(SCC/100) [32] to achieve normal distribution. Skewness (0.63), kurtosis (0.48) and visual inspection of SCS distribution confirmed the normality of the transformed variable.

### 2.3. Feed Sampling and Analysis

Three replicates of total mixed ration were sampled concomitantly with the two test days of the BF period, for a total of 6 samples. Moreover, three grass replicates (0.5 kg from 1 m^2^) were sampled from the pasture concurrently with the five test days of the EG period, for a total of 15 samples, and three replicates were sampled alongside the three days of the MLG period, resulting in 9 samples. Both feed and grass samples were weighed, dried for 120 h at 60 °C and milled through a 1 mm screen. Dried samples were weighed and analysed in the laboratory of the Breeders Association of Veneto Region for contents of dry matter (DM), crude protein, ether extract, fiber, total ash, neutral detergent fibre (NDF), acid detergent fibre (ADF) and acid detergent lignin (ADL) by NIRSystems 5000 (FOSS Analytical A/S). Hemicellulose content was calculated as the difference between NDF and ADF, and cellulose content was calculated as the difference between ADF and ADL (Table 1).

### 2.4. Data Editing and Statistical Analysis

The initial dataset comprised 658 test day records from 80 cows. Due to the low number of animals and test days, HO cows were removed from the dataset. Additionally, cows with only one test day record were discarded. Days in milk (DIM) were restricted to be between 5 and 560 days, and parity from 1 to 8. Records with MY < 2 kg/milking were discarded. Within breed, samples exceeding three standard deviations from the mean of each trait were considered as outliers and set as missing values. After editing, the dataset comprised 616 test day records from 52 SI and 19 CR cows; each individual cow was considered as an experimental unit of the present study.

In order to assess sources of variation of the studied traits, a linear mixed model was implemented in SAS software v. 9.4 (SAS Institute Inc., Cary, NC, USA) using the GLIMMIX procedure. The model was as follows:

y*_ijklm_* = μ + period*_i_* + breed*_j_* + stage*_k_* + parity*_l_* + cow*_m_*(breed*_j_*) + e*_ijklm_*,

where y*_ijklm_* is the dependent variable (milk yield, composition trait or health-related trait); μ is the overall intercept of the model; period*_i_* is the fixed effect of the *i*th period of sampling (*i* = BF, EG, MLG); breed*_j_* is the fixed effect of the *j*th cow breed (*j* = CB, SI); stage*_k_* is the fixed effect of the *k*th class of stage of lactation of the cow (*k* = 1 to 8, where the first class is 5 to 45 DIM, followed by 7 classes of 45 DIM each, and the last class including DIM > 315 days); parity*_l_* is the fixed effect of the *l*th class of parity of the cow (*l* = 1 to 6, with the last class including parities 6 to 8); cow*_m_*(breed*_j_*) is the random effect of the *m*th cow nested within the *j*th breed; and e*_ijklm_* is the random residual term. The breed effect was tested on the cow within breed variance. Interactions between fixed effects were preliminary tested, but they were not significant in explaining the variability of the studied traits and thus removed from the final model. Pearson correlation coefficients (*r*) among residuals of milk yield, composition and health-related traits obtained from the previous model were estimated. Differences between least square means of the fixed effects were tested using Tukey’s *post hoc* test (*p* < 0.05).

## 3. Results and Discussion

### 3.1. Descriptive Statistics and Sources of Variation

Milk yield averaged 13.53 kg/milking (Table 2), which was slightly greater than the average MY reported by Visentin et al. [33] and Franzoi et al. [34] for SI cows farmed in single-breed herds in the northeast of Italy. Such greater value may arise from the composition and management of the herd involved in the present trial, which comprised SI (73% of the herd, after edits) and CR cows (27% of total herd, after edits). The means of fat, protein, casein and lactose were 3.57, 3.46, 2.73 and 4.85%, respectively (Table 2). Among the major milk constituents, fat and lactose exhibited the greatest and the lowest coefficients of variation (21.00 and 4.01%, respectively). In general, the milk solid content observed in the present study was lower than that reported in the literature for multibreed herds of SI, HO and Brown Swiss cows, with particular regard to the fat percentage [33]. This may be due to the relatively high MY of the cows involved in the present study, which in turn may be related to milk solid dilution. Among health-related milk traits, the FPR ranged from 0.55 to 1.67. The indicators of udder health status, namely, SCS, DSCC and EC, averaged 2.81, 65.46% and 870.63 mS, respectively, with coefficients of variation that ranged from 8.77% (EC) to 60.42% (SCS; Table 2). Milk urea averaged 20.71 mg/dL, which was close to the value observed in the milk of dual-purpose Aosta Red Pied cows [3]. The BHB averaged 0.079 mmol/L and exhibited a wide range (0.01 to 0.22 mmol/L) and a large coefficient of variation (44.57%).

Results from the analysis of variance of MY, milk composition and health-related milk traits are summarized in Table 3. The period of sampling, DIM and parity were significant in explaining the variation of MY, fat, protein and casein content (*p* < 0.05). The lactose content varied significantly in relation to the stage of lactation (*p* < 0.001) and period of sampling (*p* < 0.05), whereas it was not affected by breed and parity. Nevertheless, a greater concentration of lactose was estimated for primiparae (4.85%) compared to multiparae (4.80%), in accordance with the review of Costa et al. [35]. Regarding the health-related traits, the period of sampling was significant for all indicators (*p* < 0.05), with the exception of SCS, and the stage of lactation was significant for FPR, SCS, EC and MU (*p* < 0.001). In general, breed was the least important fixed effect to explain the variability of the studied traits, being significant only for MU (*p* < 0.01; Table 3). The random effect of the cow nested within breed accounted for more than 50% of the total phenotypic variance of protein, casein and lactose content, SCS, DSCC and EC. The same random effect accounted for a lower proportion of phenotypic variance in the case of fat (15.36%), FPR (16.85%) and MU (16.37%; Table 3).

### 3.2. Correlations between Milk Yield, Milk Composition and Health-Related Traits

Pearson correlation coefficients between the residual terms of the studied traits are shown in Table 4. In addition to the strong (and expected) correlations between protein and casein and between fat and FPR, all other relationships were moderate to low, with the overall strongest associations between health-related milk traits and milk composition. In this view, SCS was unfavourably associated with MY (*r* = −0.27, *p* < 0.001) and lactose content (*r* = −0.41, *p* < 0.001). The decrease in milk production in cows with high somatic cells is well documented in the literature, as it is an indicator of mammary infection [36]. A moderate and negative association between SCS and lactose was observed also by Lindmark-Mänsson et al. [37] and Roveglia et al. [38]. In the present study, a weak unfavourable association between DSCC and lactose content was observed (*r* = −0.14, *p* < 0.001; Table 4), which is consistent with previous findings of Bobbo et al. [39] who reported a weak correlation (*r* = −0.05) between DSCC and lactose content in Italian HO. In general, a decrease in lactose content in the milk of cows with subclinical or clinical mastitis, which is often characterized by greater SCS, can be explained both by increased milk barrier permeability and impaired synthetic ability due to damage to the mammary tissue [40,41]. Additionally, lactose content and EC were moderately negatively correlated (*r* = −0.40, *p* < 0.001); this relationship was discussed by other authors [42,43], who observed that a decrease in lactose content together with an increase in EC and SCC represent complementary proxies for the detection of mastitis at the individual level. The negative correlation between EC and fat content observed in the present study (*r* = −0.36, *p* < 0.001) agrees with Mabrook et al. [44], who studied EC in different commercial milk samples. Milk urea was positively correlated with fat content (*r* = 0.21, *p* < 0.001) and negatively with lactose content (*r* = −0.16, *p* < 0.001; Table 4). Stoop et al. [45] reported an even weaker positive association between MU and fat (*r* = 0.07) as well as a weaker negative association between MU and lactose (*r* = −0.06). The direct association of BHB with fat (*r* = 0.16, *p* < 0.001), and the inverse association with protein (*r* = −0.13, *p* < 0.01) and casein (*r* = −0.17, *p* < 0.001) observed in the present study was discussed also by Kayano and Kataoka [46], who reported that high levels of milk ketone bodies are associated with an increased fat content and have a detrimental effect on protein concentration.

### 3.3. Correlations between Health-Related Traits

Pearson correlation coefficients between the residual terms of health-related traits in milk are reported in Table 4. The fat to protein ratio was positively associated with SCS (*r* = 0.20, *p* < 0.001), suggesting that an altered metabolic status in terms of an increased FPR may represent a risk factor for the onset of udder diseases and vice versa. Widing et al. [47] reported that both the increase and decrease in FPR resulted in a higher risk of mastitis. Indeed, an increased SCS caused by contagious pathogens results in decreased FPR (negative association between traits); on the other hand, an increased SCS related to environmental pathogens leads to an increased FPR (positive association between traits), which is likely the case of the present study. The fat to protein ratio was positively correlated with BHB (*r* = 0.21, *p* < 0.001) and MU (*r* = 0.21, *p* < 0.001; Table 4), suggesting that an increased FPR could be associated with an increased risk of hyperketonaemia [46] and an altered nitrogen metabolism. A moderate positive correlation of 0.48 (*p* < 0.001) was observed between DSCC and SCS, confirming the findings of Bobbo et al. [39] who estimated a correlation of 0.66 between SCS and DSCC in Italian HO and concluded that these two traits provide different insights into udder health status. Weak positive correlations were estimated between EC and SCS (*r* = 0.18, *p* < 0.001), and between EC and DSCC (*r* = 0.12, *p* < 0.01; Table 4). The role of milk EC as an indicator trait to screen for mastitis has been reported in the literature [48,49]. Indeed, changes in the ionic composition of mastitic milk, which is characterized by augmented SCC levels, lead to an increase in the udder tissue EC. According to Ebrahimie et al. [43], EC is among the most reliable traits that can be used in dairy farms equipped with in-line sensors to monitor udder health. Electrical conductivity was moderately negatively correlated with FPR (*r* = −0.39, *p* < 0.001), MU (*r* = −0.21, *p* < 0.001) and BHB (*r* = −0.28, *p* < 0.001). The negative association between EC and FPR reflects the relationship between EC and fat content (Table 4). The decrease in milk EC as the fat content and FPR increase is due to the size of the fat globules. Indeed, more than 97% of total fat is composed by large globules with a thin nonconductive membrane, which hamper the conductance by occupying space in the medium and by hindering ion mobility [44]. Our findings suggest that both increased MU, which indicates an altered nitrogen metabolism, and BHB, an indicator of ketosis, are associated with a reduced milk EC. A high BHB concentration in milk seems to be a gateway condition for other diseases, such as mastitis, as confirmed by the positive, although weak, correlation (*r* = 0.17, *p* < 0.001) with SCS, and to be related to an imbalanced nitrogen metabolism, in particular with an increased MU content (*r* = 0.21, *p* < 0.001).

### 3.4. Effect of Grazing on Milk Yield and Composition

Cows sampled during the BF period had an average DIM of 175 ± 115 days, whereby during the grazing periods, the average DIM was 171 ± 109 days and 172 ± 112 days for EG and MLG, respectively.

The least squares means of MY and composition traits according to the transition from BF to summer grazing of the herd are reported in Table 5. When the herd was kept indoor before the onset of the grazing season, cows produced more milk (14.60 kg/milking). Despite the energy supplementation provided at the milking sessions, a progressive reduction in MY [50], accompanied by a reduction in milk fat content, was estimated in the MLG period (Table 5). This was also highlighted in the study of Leiber et al. [51], who observed a reduction in MY concurrently with the beginning of highland pastures, primarily due to a change in cows’ diet, which is characterized by a lower energy content compared to feed offered during BF. On the other hand, the reduction in fat content did not corroborate findings of Leiber et al. [52], who investigated the effect of highland grazing on milk composition and coagulation properties. Such decreasing trend may be related to the pasture composition, in which the hemicellulose and ADL components increased by 11.40 and 5.20%, respectively, across the two grazing periods (Table 1). After an initial increase in protein and casein contents in the milk collected in the first week of grazing, their levels progressively increased throughout the summer grazing period (Table 5), mirroring the variation of crude protein in pasture, which peaked in EG (14.71%; Table 1). Lactose levels were constant across the studied period (Table 5).

### 3.5. Effect of Grazing on Health-Related Milk Traits

The least squares means of milk health-related traits for BF, EG and MLG are reported in Table 5. The fat to protein ratio showed the greatest value in late spring (1.09) when the herd was still barn farmed, and then it decreased progressively during the grazing period with the lowest value in late summer (MLG, 1.00). Such a decline was mostly associated with the same decreasing trend registered for milk fat content throughout the studied period.

Summer grazing had no effect on the SCS, which was constant throughout the three periods (Table 5). This is apparently in contrast with findings of Niero et al. [3], who observed an increased SCS content in the milk of Aosta Red Pied cows during extensive grazing, compared to the milk obtained by the same animals during the indoor farming. In that case, the greater SCS was attributable to the (i) stress exhibited by animals as a consequence of transhumance; (ii) harsh climatic conditions of alpine highland pastures; (iii) higher frequency of injuries of the mammary gland exposed to steep slopes; and (iv) lower hygiene standard of milking procedures, performed on pastures through a mobile milking parlour. Cows involved in the present study were not subjected to any of these events, since they were not exposed to the stress determined by transhumance, grazed sloping ground pastures adjacent to the barn and were milked according to the same procedures used during the BF period. For all tested periods, DSCC was always below 70%, with the only exception of the MLG period (70.22%; Table 5), which was close to the threshold proposed by Zecconi et al. [53] to identify subclinical mastitis. The results of the present study suggest only a slight deterioration of mammary health status at the end of the summer season (i.e., MLG), mostly in terms of udder susceptibility rather than the onset of overt mastitis [29]. As for DSCC, comparison with the literature is difficult since, to the authors’ knowledge, this is the first study that has investigated DSCC before and during grazing. A sharp increase in EC was observed in the milk of cows in the MLG period (Table 5). Based on these results, and in agreement with information provided by the least squares means of DSCC, animals at the end of the grazing season were likely more susceptible to mastitis compared to the BF, EG and MLG periods [54].

A sharp decrease in MU was reported during the grazing periods (Table 5). This is allegedly associated with the low protein content measured in the grass bunches sampled in the same test days (Table 1). Such a low protein content could mirror the management of the rotational grazing scheme adopted by the farm. According to this system, cows grazed on different pasture plots from the onset of the grazing season up to the mid-summer season (i.e., MLG). Afterwards, and up to late summer, the animals made a second rotation round on the same pasture plots, which, meanwhile, underwent fertilization with farm manure. Overall, the concentration of MU observed in the present study was lower than the thresholds proposed by Raboisson et al. [55], who identified the appropriate MU content associated with cows’ pregnancy or conception rate. Therefore, it is likely that grazing in the present study did not have detrimental effects on cow fertility.

Levels of BHB were relatively constant, with small variations across sampling periods. The greatest BHB value registered at the MLG period (0.085 mmol/L) was likely due to the decrease in the energy density of the feeding substrate available for the animals (Table 1). Findings of the present study agree with those reported by Berge et al. [56], who observed a slightly greater frequency of hyperketonaemia in systems in which cows were on pastures rather than housed indoor. Such differences are likely related to the fact that animals on pastures are more difficult to monitor in terms of specific nutritional needs and health status [27].

## 4. Conclusions

Summer grazing is a management strategy widely adopted in low-input dairy systems located in the mountains to maintain herd profitability, preserve the environment and increase milk value. Results revealed that the transition from BF to summer grazing is associated with a reduction in milk yield along with a slight decline in milk fat and protein contents. The variation of health-related milk indicators, such as FPR, BHB, MU, SCS, DSCC and EC, across different periods of the grazing season showed that, when properly managed, pasture grazing does not have any detrimental effects on udder health. Therefore, a well-managed grazing practice can be a viable strategy for small dairy herds operating in the mountains to reduce feeding costs by exploiting available forage without impairing cows’ health. More effort, however, should be made to expand the sample size, by including data collected from different farms located in the Alps, in order to increase the applicability of the results obtained in the current case report.

## Figures and Tables

**Table 1:** Composition of total mixed ration and grass during barn farming and early and mid-late grazing.

Component ^1^	Total Mixed Ration		Grass			
	Barn Farming (n = 6)		Early Grazing (n = 15)		Mid-Late Grazing (n = 9)	
	Mean	SD ^2^	Mean	SD ^2^	Mean	SD ^2^
Moisture, %	40.50	1.33	78.13	2.50	77.21	3.61
Chemical composition, % of dry matter						
Starch	17.21	2.45	-	-	-	-
Crude protein	13.18	0.42	14.71	1.97	13.98	1.35
Ether extract	2.29	0.09	2.44	0.17	2.47	0.27
Fibre	21.35	1.50	23.07	2.46	27.55	2.72
Total ash	8.81	0.70	5.52	0.96	5.41	0.57
NDF	35.01	2.23	47.86	3.87	54.84	4.33
ADF	22.09	1.06	27.07	2.84	31.68	2.33
Hemicellulose	12.92	1.43	20.79	2.41	23.16	2.06
Cellulose	19.22	1.50	23.78	2.40	28.21	2.60
ADL	2.87	0.25	3.29	0.73	3.46	0.37

^1^ NDF: neutral detergent fibre; ADF: acid detergent fibre; ADL: acid detergent lignin. ^2^ SD: standard deviation.

**Table 2:** Descriptive statistics ^1^ of milk yield, composition and health-related traits.

Trait ^2^	N	Mean	SD	CV, %	Min	Max
Milk yield, kg/milking	608	13.53	4.63	34.18	2.00	27.50
Milk composition, %						
Fat	584	3.57	0.75	21.00	2.05	6.36
Protein	607	3.46	0.34	9.76	2.54	4.44
Casein	606	2.73	0.27	10.04	1.95	3.46
Lactose	610	4.85	0.19	4.01	3.76	5.30
Health-related traits						
FPR, units	581	1.03	0.19	18.51	0.55	1.67
SCS, units	605	2.81	1.70	60.42	−1.06	8.76
DSCC, %	609	65.46	14.67	22.41	23.80	94.30
EC, mS	603	870.63	76.36	8.77	674.40	1127.90
MU, mg/dL	609	20.71	4.54	21.90	5.70	33.20
BHB, mmol/L	590	0.079	0.035	44.57	0.01	0.22

^1^ SD: standard deviation; CV: coefficient of variation; Min: minimum; Max: maximum. ^2^ FPR: fat to protein ratio; SCS: somatic cell score, calculated as SCS = 3 + log_2_(SCC/100), where SCC is somatic cell count; DSCC: differential somatic cell count, calculated as the ratio of neutrophils plus lymphocytes to total milk SCC; EC: electrical conductivity; MU: milk urea; BHB: β-hydroxybutyrate.

**Table 3:** F-values and significance of fixed effects included in the model for milk yield, composition and health-related traits.

Trait ^1^	Fixed Effects				Random Effect	RSD ^3^
	Period	Breed	Days in Milk	Parity	σ^2^_cow(breed)_, % ^2^	
Milk yield, kg/milking	18.12 ***	0.21	8.12 ***	7.68 ***	50.96	2.50
Milk composition, %						
Fat	20.09 ***	0.34	15.56 ***	2.85 *	15.36	0.57
Protein	56.85 ***	0.09	16.56 ***	4.13 **	56.89	0.16
Casein	65.02 ***	0.02	16.95 ***	4.32 ***	56.96	0.13
Lactose	3.28 *	0.01	4.54 ***	1.34	59.47	0.13
Health-related traits						
FPR, units	9.77 ***	0.01	3.79 ***	1.06	16.85	0.17
SCS, units	2.36	1.38	5.76 ***	5.14 ***	60.35	1.02
DSCC, %	7.99 ***	1.14	1.37	2.95 *	52.11	9.70
EC, mS	83.20 ***	0.95	6.38 ***	6.68 ***	65.60	43.26
MU, mg/dL	13.09 ***	10.11 **	8.36 ***	0.44	16.37	3.86
BHB, mmol/L	3.24 *	0.10	0.57	1.44	41.41	0.03

^1^ FPR: fat to protein ratio; SCS: somatic cell score, calculated as SCS = 3 + log_2_(SCC/100), where SCC is somatic cell count; DSCC: differential somatic cell count, calculated as the ratio of neutrophils plus lymphocytes to total milk SCC; EC: electrical conductivity; MU: milk urea; BHB: β-hydroxybutyrate. ^2^ σ^2^cow(breed): proportion of total variance accounted by cow nested within breed effect. ^3^ RSD: residual standard deviation. * *p* < 0.05; ** *p* < 0.01; *** *p* < 0.001.

**Table 4:** Pearson correlation coefficients between the residuals estimated by the model for milk yield, composition and health-related traits.

Trait ^1^	Milk Composition				Health-Related Traits					
	Fat	Protein	Casein	Lactose	FPR	SCS	DSCC	EC	MU	BHB
Milk yield	0.02	−0.17 ***	−0.11 **	0.20 ***	0.05	−0.27 ***	0.05	−0.11 **	0.02	−0.08
Milk composition										
Fat		0.10 *	0.19 ***	−0.06	0.94 ***	0.23 ***	−0.03	−0.36 ***	0.21 ***	0.16 ***
Protein			0.98 ***	−0.01	−0.20 ***	0.11 **	−0.02	−0.09 *	0.03	−0.13 **
Casein				0.10 *	−0.11 **	0.07	0.00	0.02	0.02	−0.17 ***
Lactose					−0.06	−0.41 ***	−0.14 ***	−0.40 ***	−0.16 ***	−0.08
Health-related traits										
FPR						0.20 ***	−0.02	−0.39 ***	0.21 ***	0.21 ***
SCS							0.48 ***	0.18 ***	0.06	0.17 ***
DSCC								0.12 **	0.03	0.04
EC									−0.21 ***	−0.28 ***
MU										0.21 ***

^1^ FPR: fat to protein ratio; SCS: somatic cell score, calculated as SCS = 3 + log_2_(SCC/100), where SCC is somatic cell count; DSCC: differential somatic cell count, calculated as the ratio of neutrophils plus lymphocytes to total milk SCC; EC: electrical conductivity; MU: milk urea; BHB: β-hydroxybutyrate. * *p* < 0.05; ** *p* < 0.01; *** *p* < 0.001.

**Table 5:** Least squares means (with standard errors) of milk yield, composition and health-related traits across periods of sampling.

Trait ^1^	Barn Farming	Early Grazing	Mid–Late Grazing
Milk yield, kg/milking	14.60 (0.44) ^a^	13.60 (0.41) ^b^	12.73 (0.42) ^c^
Milk composition, %			
Fat	3.71 (0.07) ^a^	3.66 (0.05) ^a^	3.33 (0.06) ^b^
Protein	3.42 (0.03) ^b^	3.53 (0.03) ^a^	3.36 (0.03) ^c^
Casein	2.68 (0.03) ^b^	2.79 (0.02) ^a^	2.65 (0.02) ^b^
Lactose	4.80 (0.03) ^b^	4.82 (0.02) ^ab^	4.84 (0.02) ^a^
Health-related traits			
FPR, units	1.09 (0.02) ^a^	1.04 (0.02) ^b^	1.00 (0.02) ^b^
SCS, units	3.37 (0.21) ^a^	3.22 (0.20) ^a^	3.10 (0.20) ^a^
DSCC, %	68.53 (1.75) ^ab^	66.40 (1.62) ^b^	70.22 (1.65) ^a^
EC, Ms	884.55 (9.60) ^b^	877.86 (9.13) ^b^	934.86 (9.23) ^a^
MU, mg/dL	21.90 (0.45) ^a^	19.85 (0.36) ^b^	19.95 (0.40) ^b^
BHB, mmol/L	0.082 (0.004) ^ab^	0.078 (0.004) ^b^	0.085 (0.004) ^a^

^a,b,c^ Least squares means with different superscripts letters within a row differ significantly (*p* < 0.05). ^1^ FPR: fat to protein ratio; SCS: somatic cell score, calculated as SCS = 3 + log_2_(SCC/100), where SCC is somatic cell count; DSCC: differential somatic cell count, calculated as the ratio of neutrophils plus lymphocytes to total milk SCC; EC: electrical conductivity; MU: milk urea; BHB: β-hydroxybutyrate.

## Data Availability

The data presented in this study are available on request from the corresponding author.
